# Retraction: Effect and mechanism of inhibition of PI3K/Akt/mTOR signal pathway on chronic neuropathic pain and spinal microglia in a rat model of chronic constriction injury

**DOI:** 10.18632/oncotarget.28848

**Published:** 2026-04-17

**Authors:** Jian-Rong Guo, Huan Wang, Xiao-Ju Jin, Dong-Lin Jia, Xun Zhou, Qiang Tao

**Affiliations:** ^1^Department of Anesthesiology, Gongli Hospital, Second Military Medical University, Shanghai 200135, P.R. China; ^2^Department of Anesthesiology, Yijishan Hospital, Wannan Medical College, Wuhu 241001, P.R. China; ^3^Pain Department, Peking University Third Hospital, Beijing, 100191, P.R. China

**This article has been retracted:** Oncotarget has completed its investigation of this paper. It was determined that multiple instances of image duplication and overlap with unrelated papers were found. Specifically, we identified the following issues: Figures 2B and 2C (immunohistochemistry (IHC) assay for OX-42 in marrow of rats in sham and CCI groups) overlap with Figures 6C and 6B (IHC for OX-42 in the wortmannin- and DMSO-treated groups), respectively. In addition, Figure 6A (IHC of control group) overlaps with Figure 6A in a concurrently submitted unrelated paper from different institution [1], and Figure 6B (IHC of DMSO-treated group) overlaps with IHC images in Figure 7A of [2] and Figure 4D of [3]. Moreover, Figure 7A panel illustrating expression of GFAP in the wortmannin-positive control overlaps with the image of the DMSO-treated group.

The authors were contacted to provide comments and raw data regarding these concerns. While they requested a correction, they failed to provide the original supporting data or a sufficient explanation for the overlaps.

Due to the volume of figure duplications and the absence of original data, the editorial decision has been made to retract the paper. The authors have been informed of this decision.

Original article: Oncotarget. 2017; 8:52923–52934. 52923-52934. https://doi.org/10.18632/oncotarget.17629
